# Supplementary material for: Concomitant Calcium Channelopathies Involving CACNA1A and CACNA1F: A Case Report and Review of the Literature
Source: Genes (Basel). 2023 Feb 3;14(2):400. doi: 10.3390/genes14020400 (PMC9956337; doi:10.3390/genes14020400)
Supplement: Supplementary file 1 [file genes-14-00400-s001.zip › Supplementary Document S1.docx]

Comparison of manifestations seen in FHM1 versus SHM1

|  | **FHM1**(OMIM 141500) | **SHM1** |
| --- | --- | --- |
| **Symptoms During Hemiplegic Attacks** |  |  |
| Migraine with aura |  |  |
| Hemiplegia |  |  |
| Neurologic Manifestations |  |  |
| Coma/Altered Consciousness | + <33% |  |
| Seizure (Including Status Epilepticus) |  |  |
| Cerebral Edema | + Less Common |  |
| EEG Slowing or Spike Waves |  |  |
| Eye Abnormalities (Including Transient Blindness) |  |  |
| Encephalopathy |  |  |
| Dystonic Storms |  |  |
| Lethargy |  |  |
| Autonomic Manifestations |  |  |
| Fever (Non-Infectious) |  |  |
| Vomiting/Nausea |  |  |
| Apnea |  |  |
| Other Symptoms |  |  |
| Speech Difficulties |  |  |
| Photophobia |  |  |
| Phonophobia |  |  |
| Abnormal CSF (Including high IL-6) |  |  |
| **Symptoms Outside of Hemiplegic Attacks** |  |  |
| Movement Disorders |  |  |
| Ataxia |  |  |
| Dystonia |  |  |
| Pyramidal Signs |  |  |
| Tremor | + Less Common |  |
| Dyskinesia |  |  |
| Global Developmental Delay |  |  |
| Verbal Conditions |  |  |
| Intellectual Disability |  |  |
| Brain Atrophy |  |  |
| Paroxysmal Actions |  |  |
| Abnormal Eye Movements |  |  |
| Seizures | + Less Common |  |


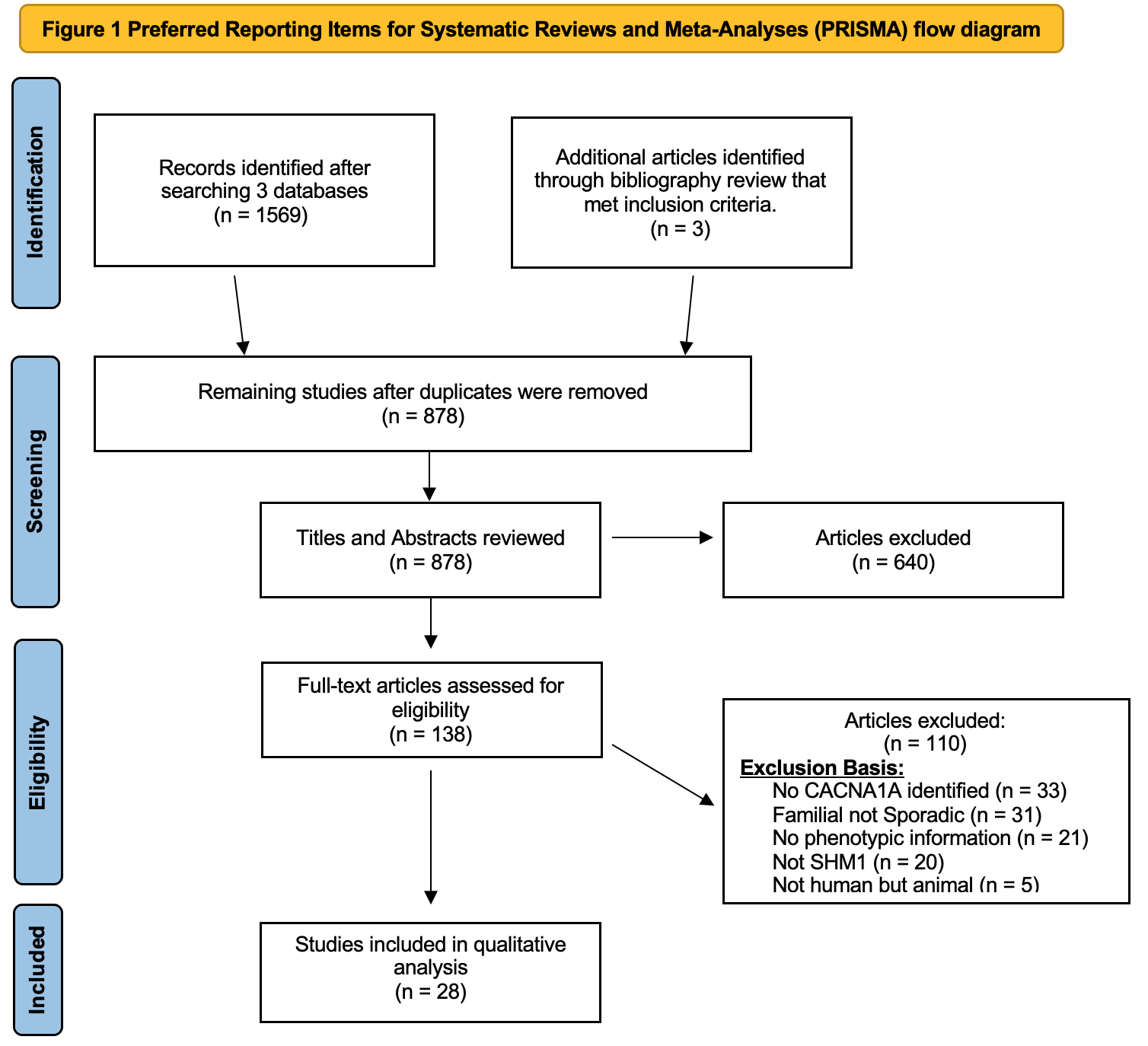


**Figure 1** PRISMA Flowchart for the systematic literature review, *CACNA1A* related phenotype-SHM1 phenotype.

| Search Strategy | SHM1-*CACNA1A* |
| --- | --- |
| Keywords | MeSH Terms |
| "CACNA1A" OR “CACNA1A protein” OR “voltage-dependent calcium channel (P-Q type)” AND "hemiplegic migraine” OR “Migraine, hemiplegic” | [Calcium Channels (only to be used in conjunction with “AND CACNA1A”) AND Hemiplegia (only to be used in conjunction with "AND Migraine") |
| "Phenotype" OR "character*" OR "present*" OR "trait" OR "manifest*" | Phenotype |
| "Human" OR "Carriers" OR "Patients" OR "male" OR "female" | Human OR heterozygote OR Patient OR male OR female |

Search Strategies for SHM1-*CACNA1A* Systematic Literature Review

| **Study Type Distribution** |  | Included Studies (N=44) |
| --- | --- | --- |
|  | N | (%) |
| Study Type |  |  |
| Case Study | 21 | 47.7% |
| Cohort Study | 11 | 25.0% |
| Cross-Sectional Study | 2 | 4.5% |
| Review with Case Study | 2 | 4.5% |
| Collective Case Study | 2 | 4.5% |
| Review | 2 | 4.5% |
| Multicenter Study | 2 | 4.5% |
| Case Report + Functional Study | 1 | 2.4% |
| Follow Up Case Study | 1 | 2.4% |

SHM1 patient demographics.

|  |  |
| --- | --- |
| **Sex** | **No (%)** |
| Female | 26 (54.2%) |
| Male | 20 (41.7%) |
| Unknown | 2 (4.1%) |
| **Age Group** |  |
| <10Y | 11 (22.9%) |
| 11Y to <20Y | 19 (39.6%) |
| 21Y to <30Y | 1 (2.1%) |
| 31Y to <40Y | 5 (10.4%) |
| 41Y to <50Y | 1 (2.1%) |
| 51Y to <60Y | 1 (2.1%) |
| Unknown | 10 (20.8%) |
