# Supplementary material for: Concomitant Calcium Channelopathies Involving CACNA1A and CACNA1F: A Case Report and Review of the Literature
Source: Genes (Basel). 2023 Feb 3;14(2):400. doi: 10.3390/genes14020400 (PMC9956337; doi:10.3390/genes14020400)
Supplement: Supplementary file 1 [file genes-14-00400-s001.zip › Supplementary Document S5.docx]

**21 months**: After prolonged vomiting, the patient became unresponsive. His breathing was shallow (Resp rate 8). He was tachycardic (HR 155) with mild twitching of his left foot. He was rushed to ER, given anti epileptics, intubated, a CT and lumbar puncture were performed and then he was transferred to local children’s hospital for care. Abnormal Labs and CSF included acidosis plus high glucose, high WBCs, high CRP, low calcium, low bilirubin, and lymphocytic pleocytosis. At the Children’s Hospital, EEG demonstrated focal spikes and MRI showed mild edema suspicious of encephalitis. Patient was started on an antiviral; however, CSF was negative for infectious agents and the antiviral was stopped. Patient was extubated with a quick recovery and discharged within 5 days of the attack.

**2 Years 2 Months**: After possible food trigger or mold exposure, the child awakened in the night vomiting and choking. He became unresponsive with apnea and was rushed to the ER. At the ER, he suffered a focal seizure and was given Ativan. He was tachycardic and his abnormal labs included acidosis plus high glucose, high WBCs, and low calcium. The patient was transferred to the children’s hospital and given Solumedrol 10 mg IV per the FPIES protocol but developed giant hives. The EEG was normal as well as additional genetic testing to rule out Fragile X. The patient was discharged within 4 days.

**2 Years 4 Months**: Another episode of extreme vomiting with unresponsiveness and the patient was taken to the ER. Again, the subject was tachycardic and his labs revealed: acidosis, high glucose, high WBCs, and low calcium. The patient received fluids and Solumedrol 10mg IV. Within minutes, the child became unresponsive, yet within a few hours the patient was discharged. Overnight, the subject worsened with a high fever (103^O^) and experienced paralysis on the right side (1^st^ time). The child was taken to the children’s hospital ER. Labs were drawn and a lumbar puncture and MRI was performed. The patient’s mental status worsened becoming catatonic. He was seen by pediatric neurology and infectious disease, who prescribed azithromycin. All tests were negative for infectious agents, but revealed a high CRP, an abnormal MRI with edema and diagnosis of Dandy-Walker variant. His EEG was irregular with slowing and spike waves. On day 3, the patient’s mental status improved but he developed scrotal swelling as well as inflammation in the knee joints. By day 4, the fever began to subside, but patient suffered a skin reaction to the IV azithromycin, and it was discontinued. The child returned to near-baseline and was discharged on day 5.

**3 Years 9 Months**: After a food trial, the patient was up in the middle of the night, suffered a seizure and fell out of bed (20 inches onto a carpeted floor). He was unresponsive, vomited and was rushed to the ER. Once at the ER, he experienced a left sided focal seizure and was given Ativan 1 mg. His labs revealed severe acidosis and a very high WBC, high glucose, and low calcium. He was sedated and intubated. He developed a high fever of 103^O^ and was transferred to local children’s hospital, where they performed a lumbar puncture which revealed lymphocytic pleocytosis. Then, additional labs were drawn to investigate infectious agents while beginning the patient on acyclovir, under suspicion of encephalitis. Within 24 hours, the patient developed left-sided paralysis. His EEG suggested subclinical seizures and he was started on Keppra 15 mg/kg. On day 2, his MRI was abnormal, similar to the findings from 2012. His lumbar puncture and infectious panel were negative. A high fever continued and was unresponsive to acetaminophen and ibuprofen. CRP and Segs continued to remain high. Pediatric Neurology requested testing for mitochondrial disorders, highly convinced the patient suffered from MELAS (Mitochondrial Encephalopathy Lactic Acid Syndrome). By day 3, patient was extubated but struggled to come out of sedation. He stayed on continuous EEG monitoring, which revealed slowing. The child suffered a brief focal seizure in left hand and was given Ativan 1 mg on day 4. Also, Keppra was increased to BID, and the fever continued to decrease. The patient was discharged on Day 5, but unlike other episodes, the left sided weakness had not fully resolved, and patient remained on the antiepileptic therapy.

**4 Years 5 Months:** After a four-day food trial, the patient vomited at 1am and remained awake until suffering a tonic-clonic seizure at 4:45am. He became unresponsive with apnea and was rushed to the ER. Once at the ER, the patient continued to seize and was given Ativan 1 mg IV. He appeared to be in pain and was given morphine. The patient continued to seize was given Fosphenytoin 250 mg IV and transferred to the PICU. Once in the PICU, the patient suffered a 45-minute tonic-clonic seizure and was given multiple doses of Ativan 1 mg IV and then intubated. His labs revealed high glucose, high WBCs, low calcium, high CPK, high lactic acid. A high fever developed within the hour of the last seizure and was unresponsive to acetaminophen and ibuprofen. The patient was started on a sepsis protocol of Vancomycin and Rocephin against family’s request. An EEG was performed. On day 2, the MRI showed abnormal results and the EEG was reattached due to right-sided paralysis which revealed slowing. The patient’s face began to swell, the family suspected it was from the antibiotics, so Benadryl and Lasix were given. Dexamethasone was administered to prepare for extubation. Fever remained unresponsive to treatment, 105^O^. The family met with genetics, who ordered Defects of Glycosylation and Inborn Errors of Metabolism Testing. Both tests came back negative. By day 3, the patient was extubated, and the EEG was removed due to no seizure activity in 48 hours. The patient’s abnormal labs continued (first time hourly labs were measured) including high glucose, high WBCs, low creatinine, low calcium, high CRP. Antibiotics were stopped due to all labs were negative for infection. The fever continued and the patient began drinking. On day 4, the patient demonstrated withdrawal symptoms and remained unresponsive and reattached to EEG. However, he was able to eat and drink. Due to continued altered consciousness, a lumbar puncture was administered for further infectious investigation. One neutrophil and high glucose was noted on the CSF report but showed no signs of infection. By day 6, the patient began to become responsive, smiling without any eye contact. He received physical therapy and was exposed to airborne peanut butter. Within 90 seconds of exposure, he suffered a tonic clonic seizure. Ativan 1 mg was administered, and the patient was reconnected to the EEG. The child suffered another seizure 9 hours later and his team restarts Fosphenytoin 250 mg IV. On Day 7, the ophthalmologist confirmed that the patient has lost his vision although the optic nerve looked normal. Rheumatology believes that the patient suffers from an auto-inflammatory disorder, ran T & B Cell and Immunoglobulin levels as well as Behcets Disease and wanted him to go to the NIH for diagnosis. The patient is discharged on Day 11 and still has impaired vision. His eyes react to light, and they believe he may have some distance vision.

**7 Years 7 Months**: 36 hours after exposure to high altitude (3500 ft) crossing the Smoky Mountains, the patient developed transient facial paralysis which resolved. The next morning, he began vomiting and developed right-sided paralysis. Within an hour, he began a tonic-clonic seizure and was rushed to the local ER in status epilepticus. He was given Ativan 1mg IV and Fosphenytoin 250mg IV, but the seizure continued for 2 hours. He was tachycardic and his labs revealed high glucose, high WBCs, and low calcium. He developed a very high fever which was resistant to acetaminophen and ibuprofen. The patient was transferred to a different children’s hospital north of their home. He was taken to PICU and hooked up to continuous EEG with no seizure activity apparent. Day 2, the hospital pediatric neurologist suspected Alternating Hemiplegia of Childhood (AHC) and requested 3 genes be reviewed by the investigator at the NIH. The patient continued to be febrile with no response to NSAIDs and abnormal labs included high WBCs, high CRP, and high ESRs, high ammonia, low bilirubin, low potassium, and low calcium. On day 3, the patient remained very sedated, but his eyes became equal and reactive although significantly dilated with horizontal nystagmus. His labs show metabolic acidosis. On day 4 a follow up BMP showed worsening metabolic acidosis and suspicion of Renal Tubular Acidosis (RTA). He was started on IV bicarbonate. Labs continue to be abnormal with high ESR, high CRP, low sodium, low potassium, and low calcium. Additionally, a systolic murmur was identified, and an echo was ordered to rule out endocarditis. On day 5, it was determined that metabolic acidosis was improving, and RTA seemed unlikely, however, the patient received an oral dose of bicarbonate. The endocardiogram was negative for endocarditis. On day 6, nephrology discontinued bicarbonate and believed RTA was transient potentially due to Toradol the patient received. On day 8, the patient’s fever is improving while hemiplegia remains stagnant. The patient is still quite sedated with very low tone and struggling to eat and drink. The subject is given a CT instead of MRI due to past issues with sedation. Abnormal findings on the CT includes extensive cytotoxic edema in the left cerebral hemisphere. That evening the patient suffered a seizure that required two doses of Ativan 1 mg, 5 minutes apart and a loading dose of Fosphenytoin 250mg to resolve. The patient was transferred to PSCU for additional monitoring. On day 9, the child was transferred back to the pediatrics floor with IV fluids discontinued. Lab abnormalities were resolving. Again, patient has a loss of vision. Confirmation was received from the NIH that the patient had a novel, de novo *CACNA1A* variant which at that time had been linked to Alternating Hemiplegia of Childhood (later it would be separated into Sporadic Hemiplegic Migraine type 1 while AHC was linked to *ATP1A3*). The patient needed to re-test for clinical diagnosis with a CLIA-certified lab. The patient suffered another seizure less than 12 hours of being transferred to the pediatrics floor (an old section of the hospital with potential mold, a known trigger for the patient). He seized for 45 minutes, not responding to 2 doses of Ativan 1.5 mg, 1 dose of Ativan 3 mg, followed by a loading dose of Fosphenytoin, 250 mg. The patient finally stopped seizing with the addition of a loading dose of Phenobarbital and was transferred back to PICU and attached to continuous EEG monitoring, showing burst suppression over the left hemisphere. On day 10, a PICC line was placed, and the patient was sent for an MRI since he was already significantly sedated. The MRI confirmed the abnormal results seen in the CT and no stroke. On day 11, the patient was so sedated that he needed a foley catheter to be placed and IV fluids resumed. The phenytoin levels were above the acceptable range and his Fosphenytoin was held. The EEG showed no seizure activity but a slowing of 2 to 3 Hertz while the patient was awake. On day 14, neurology started the patient on Verapamil 40 mg BID (used to treat *CACNA1A* with varied results, no RCTs to confirm efficacy or safety). The patient had the foley removed and began to eat and drink again. The fever had resolved, and paralysis was greatly improved. However, telemetry showing premature ventricular contractions (PVCs- bigeminy and trigeminy) and an episode of accelerated ventricular rhythm since starting Verapamil. A 24-hour Holter is requested by cardiology. On day 15, the cardiac Holter monitor was applied and the PICC line removed. By day 16, the Holter monitor is removed and read. The patient is close to baseline and discharged on Verapamil 40mg BID.

**Minor Attacks:**

9 Years 11 Months: After potential mold exposure, the patient suffered significant vomiting followed by high fever, altered mental status- encephalopathy, and right-sided hemiplegia. He was tachycardic and abnormal labs included high glucose, high WBCs, high CRP, and low calcium. Fever of 105^O^ for 24 to 36 hours which was non-responsive to NSAIDs. The EEG demonstrated slowing on left hemisphere, but right side was normal. The patient, again, suffered loss of vision. Flash MRI showed no change since the severe attack at 2 years 4 months of age. The patient was discharged at baseline except for vision. The duration was 6 days. His vision returned approximately 14 days later.

11 Years: After mold exposure, the child experienced significant vomiting followed by high fever, altered mental status with encephalopathy, and right-sided hemiplegia. The patient experienced fever of 105^O^ for 12 hours and was non-responsive to NSAIDs. The parents kept the child at home but had to visit the ER due to urinary retention. The symptoms resolved within 4 days.

11 Years 9 Months: Without any known exposure, the patient experienced Paroxysmal Dyskinesia shortly after falling asleep. He was altered and thrashing in bed, either ballism or athetosis. He was transported to the ER where he was administered the Status Epilepticus (SE) protocol, established after diagnosis, of 3 doses of Ativan 1 mg followed by a loading dose of Fosphenytoin, 74.4 mL IVPB, and a loading dose of phenobarbital, 74.4 mL Pediatric IV, and transported to his home children’s hospital. His initial presentation included tachycardia, high glucose, and low calcium. However, due to therapeutic brain suppression, the patient never developed hemiparesis, fever, or transient blindness. His EEG did not show any seizure activity but did show diffuse encephalopathy with the right hemisphere demonstrating more cortical dysfunction. Neurology ordered another flash MRI which showed no change. He slept for 2 days and was discharged on day 3. However, he did suffer from periodic ataxia for 3 to 4 weeks post event.

12 Years: The patient suffered another Paroxysmal Dyskinesia event as he finished dinner and was taken to the ER. The movements were less severe, more like athetosis, and the ER physician only administered 2 doses of Ativan 1.5 mg and the patient was transferred to his children’s hospital. Additionally, the patient was tachycardic, but had mostly normal labs except for high glucose. While in transport approximately 6 hours after the event, he developed a fever and right-sided paralysis. The fever continued to climb to 105^O^ approximately 24 hours after the attack and persisted for 12 to 24 hours, resistant to all NSAIDs. During that time, the subject suffered from encephalopathy. His EEG showed slowing and scattered sharp waves but no seizure activity. As he regained consciousness, loss of vision was noted. The patient’s fever and hemiplegia resolved, and he was discharged on day 5. His vision returned approximately 2 weeks later.

12 Years 1 Month: The patient suffered another Paroxysmal Dyskinesia event as he was getting ready for bed. He was transported to the ER and the team followed his SE protocol (see above) and transported to the children’s hospital. His labs revealed high glucose, high WBCs, and high CPKs. Also, he was tachycardic. Again, due to brain activity suppression, the patient did not suffer from fever, hemiplegia, or loss of vision. However, the sedation was severe and took longer for the patient to awaken. He was discharged on day 5. However, he suffered from severe periodic ataxia for over a month after the event.

12 Years 2 Months: The patient had a brief episode of Paroxysmal Dyskinesia in the form of Dystonia lasting less than 30 seconds while sleeping. The parents were able to get him to return back from the altered status and “break the attack by moving his locked head, torso, and limbs”. He went back to sleep but displayed weakness on his left side and had an elevated temperature for about 6 hours in the morning. There was no loss of vision or significant hemiparesis.

12 Years 2 Months: The patient suffered another brief dystonic episode of less than 30 seconds with transient residual left-sided weakness.

12 Years 3 Months: The patient had another mild episode of Paroxysmal Dyskinesia similar to athetosis in the middle of the night, lasting less than 3 hours. This time the parents decided to try to keep him home. He developed a fever and right-sided paralysis about 6 hours after the altered movements. The fever climbed to 105^O^ about 24 hours later and remained there for approximately 24 hours without response to any mechanism to reduce it including cool compresses and ice packs under the sheets. He suffered from encephalopathy until the fever broke on day 3. His lost vision resolved about 10 days later.

12 Years 4 Months: The patient had another brief dystonic episode while sleeping that lasted about 30 seconds with left-sided weakness followed by another one approximately 2 weeks later. No long-term side effects were seen from either event.

Description of each attack suffered by the patient in chronological order.

| Minor Attacks |  |  | |  |  |  |  |
| --- | --- | --- | --- | --- | --- | --- | --- |
| Episode | Age | Duration | | Symptoms | Diagnostics | Therapeutics | Potential  Trigger |
| 1 | 5 M | <20 secs | Vomiting  Unresponsive | | Remained  Home |  | Cereal-Type  Unknown |
| 2 | 21 Months | 5 Days | Vomiting  Coma  Apnea  Intubation  Tachycardia  Seizure  Fever  Abnormal Labs  Abnormal EEG  Abnormal MRI | | Labs  CSF  EEG  MRI | Antiepileptics Acyclovir  NSAIDs | Food-Diet  Alteration |
| 3 | 22 M | <24 Hours | Vomiting  Lethargy | | Remained  Home |  | Food-Diet  Alteration |
| 4 | 2 Y 2M | 4 Days | Vomiting  Coma  Apnea  Seizure  Fever  Abnormal Labs | | Labs | Ativan  Solumedrol  NSAIDs | Food or  Airborne  Allergen |
| 5 | 2 Y 4 M | 5 Days | Vomiting  Coma  Tachycardia  Abnormal labs  Fever  Encephalopathy  Rt-Side Hemiplegia  Abnormal EEG  Abnormal MRI | | CSF  Labs  EEG  MRI | Solumedrol  Azithromycin  NSAIDs | Transdermal Vitamin B-12 |
| 6 | 3 Y 9 M | 5 Days | Vomiting  Coma  Seizure  Status Epilepticus  Intubation  Abnormal labs  Fever  Encephalopathy  Lft-side Hemiplegia  Abnormal EEG  Abnormal MRI  Potential Stroke | | Labs  CSF  EEG  MRI | Ativan  Keppra  NSAIDs | Mild Head Trauma |
| 7 | 4 Y 5M | 11 Days | Vomiting  Coma  Apnea  Seizure  Status Epilepticus  Abnormal labs  Fever  Encephalopathy  Rt-Side Hemiplegia  Abnormal EEG  Abnormal MRI  Transient Blindness | | Labs  CSF  EEG  MRI | Ativan  Fosphenytoin  Vancomycin  Rocephin  Benadryl  Lasix  Dexamethasone  NSAIDs | Food-Diet  Alteration |
| 8 | 6 Y 7 M | <6 Hours | Vomiting  Lethargy  Left-side Hemiplegia  Labs-Suspicious of  Sepsis | | ER Visit  Labs | IV Fluids | Airborne  Allergen |
| 9 | 7 Y 7 M | 16 Days | Vomiting  Coma  Seizure  Status Epilepticus  Tachycardic  Abnormal labs  Fever  Encephalopathy  Rt-side Hemiplegia  Abnormal EEG  Abnormal MRI  Horizontal Nystagmus  Transient Blindness  Renal Tubular Acidosis  Heart Murmur | | Labs  EEG  MRI | Ativan  Fosphenytoin  NSAIDs  Bicarbonate  Verapamil | High Altitude (3500 ft) |
| 10 | 9 Y 11 M | 6 Days | Vomiting  Fever  Encephalopathy  Rt-side Hemiplegia  Transient Blindness | | Labs  EEG  Flash MRI | NSAIDs | Airborne  Allergen |
| 11 | 11 Y | 4 Days  (Home) | Vomiting  Fever  Encephalopathy  Rt-side Hemiplegia  Transient Blindness | | Straight Catheter | NSAIDs | Airborne  Allergen |
| 12 | 11 Y 9 M | 3 Days | Paroxysmal Dyskinesia  (ballism)  Tachycardic  Encephalopathy | | EEG  Flash MRI | Ativan  Fosphenytoin  Phenobarbital | Mold-  Multiple strains |
| 13 | 12 Y | 5 Days | Paroxysmal Dyskinesia  (athetosis)  Tachycardic  Fever  Encephalopathy  Rt-side Hemiplegia  Transient Blindness | | EEG | Ativan  NSAIDs | Mold-  Multiple strains |
| 14 | 12 Y 1 M | 5 Days | Paroxysmal Dyskinesia  (ballism)  Tachycardic  Encephalopathy | |  | Ativan  Fosphenytoin  Phenobarbital | Mold-  Multiple strains |
| 15/16 | 12 Y 2 M | <30 secs  (2 separate episodes 2 weeks apart) | Paroxysmal Dyskinesia  (Dystonia)  Brief left-side weakness  Elevated temperature (6hrs) | |  |  | Mold-  Multiple strains |
| 17 | 12 Y 3 M | 3 Days  (Home) | Paroxysmal Dyskinesia  (athetosis)  Tachycardic  Encephalopathy  Fever  Rt-side Hemiplegia  Transient Blindness | |  | Cool Compresses  Ice packs | Mold-  Multiple strains |
| 18/19 | 12 Y 4 M | <30 secs  (2 separate episodes 2 weeks apart) | Paroxysmal Dyskinesia  (Dystonia)  Brief left-side weakness | |  |  | Mold-  Multiple strains |
| 20 | 12 Y 6 M | 2 Days  **Shortened Due to Therapeutic Protocol** | Paroxysmal Dyskinesia  (ballism)  Tachycardic  Encephalopathy  Rt-side Hemiplegia  Fever <102 for <24 hours | | Reduced Hospital stay to 36 hours | Dexamethasone IV (0.5 mg/kg/day) 3 pulses for up to 3 days | Mold-  Multiple strains |
